# You are how you eat: differences in trophic position of two parasite species infecting a single host according to stable isotopes

**DOI:** 10.1007/s00436-020-06619-1

**Published:** 2020-02-06

**Authors:** Beric M. Gilbert, Milen Nachev, Maik A. Jochmann, Torsten C. Schmidt, Daniel Köster, Bernd Sures, Annemariè Avenant-Oldewage

**Affiliations:** 1 Department of Zoology, University of Johannesburg, 524 Auckland Park, Johannesburg, 2006 South Africa; 2 Aquatic Ecology, University of Duisburg-Essen, Universitätsstr. 5, 45141 Essen, Germany; 3 Centre for Water and Environmental Research, University of Duisburg-Essen, Universitätsstr. 5, 45141 Essen, Germany; 4 Instrumental Analytical Chemistry, University of Duisburg-Essen, Universitätsstr. 5, 45141 Essen, Germany; 5 Spectrum Analytical Facility, University of Johannesburg, 524 Auckland Park, Johannesburg, 2006 South Africa

**Keywords:** Monogeneans, Cestodes, Stable isotopes, Trophic relationships, Host–parasite system

## Abstract

Parasitism is commonly recognised as a consumer strategy, although, the interaction of parasites in communities and ecosystems are generally poorly understood. As parasites are integral parts of food webs, analysis of the trophic interactions between parasites and hosts was assessed through comparison of stable isotope ratios of carbon (^13^C/^12^C) and nitrogen (^15^N/^14^N). Largemouth yellowfish (*Labeobarbus kimberleyensis*) infected with the Asian tapeworm (*Schyzocotyle acheilognathi*) were collected from the Vaal Dam. Signatures of δ^13^C and δ^15^N were assessed in host muscle and liver tissue, and cestodes using an elemental analyser coupled with an isotope ratio-mass spectrometer (EA-IRMS). Hosts were enriched by 4.1‰ in the heavy nitrogen isotope with respect to the *S. acheilognathi* and therefore occupy a higher trophic position than the parasite. Comparison of δ^13^C indicates that dietary sources of carbon in cestodes are derived from the host liver. Comparison of stable isotope signatures between *Paradiplozoon ichthyoxanthon* (another common parasite of the Largemouth yellowfish in the Vaal River) and *S. acheilognathi* showed that the monogenean was enriched by 5.3‰ in ^15^N which accounts for a difference of almost two trophic positions. Isotope differences in the host–parasite system considered indicate that differences can be related to the mode of nutrient acquisition employed by host and parasites. Cestodes, being depleted in both ^13^C and ^15^N relative to the host and monogenean (*P. ichthyoxanthon*), indicate that *S. acheilognathi* assimilates nutrients derived from the host metabolism which are released from the liver.

## Introduction

Analysis of stable isotope ratios of carbon (^13^C/^12^C) and nitrogen (^15^N/^14^N) as a method for interpreting food web architecture and energy flows based on differences in isotope signatures (Doucett et al. 1999; Thompson et al. 2005; Wada 2009; Sures et al. 2019) has become increasingly popular in ecological studies. Differences in these isotopic signatures serve as a time-integrated representation of the diet of organisms based on nutrient assimilation rather than ingestion of food materials (Fry and Sherr 1984). Generally, studies have shown that consumers are enriched in ^15^N by 3.4‰ per trophic level, and thus differences in nitrogen enrichment serve as a representation of the position of organisms in the trophic hierarchy (Minagawa and Wada 1984; Vander Zanden et al. 1997). Food sources incorporated into the diet of organisms can be determined through analysis of carbon (^13^C) signatures (Post et al. 2000). More recently, stable isotope analysis (SIA) has been applied as a means of better understanding the intricate relationship between hosts and parasites (Sabadel et al. 2016; Nachev et al. 2017; Yohannes et al. 2017; Sures et al. 2019).

Parasites are important components of ecosystems and are known to shape and modify the structure and stability of food webs (Marcogliese 2003; Poulin 2010; Nachev et al. 2017). Despite their close link with the food web, they have been somewhat neglected in studies on trophic relationships in ecosystems (Thompson et al. 2005). This has been related to the fact that inclusion of parasitism in food web studies would ultimately result in invalidation of the trophic cascade model, as inclusion of parasitism in food webs leads to looping, increased omnivory and increased chain length (see Marcogliese and Cone 1997). Despite these effects, understanding how parasites interact with host organisms is crucial to understanding the ecology of parasites (Nachev et al. 2017). The relationship between parasites and hosts has mostly been synonymised with that of a predator–prey relationship (Poulin 2010; Nachev et al. 2017). This therefore suggests that parasites should be enriched in the heavier nitrogen isotope similarly to a predator (Poulin 2010). However, unlike predatory organisms, most parasites only derive nutrients from a single host at any one time in the life history, and as such only attain a fraction of the total biomass of their hosts (Lafferty and Kuris 2002).

Studies comparing isotope signatures between hosts and parasites have indicated that enrichment of the heavier nitrogen isotope in parasites is largely linked to the mode these organisms have developed to acquire nutrients (Nachev et al. 2017). For instance, endoparasites such as cestodes and acanthocephalans which acquire nutrients through assimilation of molecules derived from the hosts’ metabolism are depleted in ^15^N isotope. Whereas, ectoparasites such as fleas (Boag et al. 1998), nymphs of *Pteronarcys biloba* (Doucett et al. 1999), ticks (Schmidt et al. 2011) and some monogeneans (Sures et al. 2019), which actively feed on host tissues are ^15^N-enriched compared to their hosts (Pinnegar et al. 2001; Deudero et al. 2002; Persson et al. 2007). In a study of 10 different species of fish hosts Deudero et al. (2002) found that cestodes and nematodes were depleted in ^15^N compared to their hosts, while copepod parasites were ^15^N-enriched. Similarly, Pinnegar et al. (2001) observed that *Schistocephalus solidus* (Cestoda) and *Hysterothylacium aduncum* (Nematoda) were ^15^N-depleted compared to their hosts. As for ectoparasites, in the same study, the authors however, observed that *Lernaeocera branchialis* (Copepoda) and *Anilocra physodes* (Isopoda) were not enriched in the heavier nitrogen isotope compared to their hosts. Persson et al. (2007) found that the cestode *Eubothrium crassum* was depleted in ^15^N. Sures et al. (2019) showed that, compared to two fish hosts infected with the monogenean *Paradiplozoon ichthyoxanthon*, parasites were ^15^N-enriched, which corroborated observations for other ectoparasites (Boag et al. 1998; Doucett et al. 1999; Deudero et al. 2002; Voigt and Kelm 2006; Schmidt et al. 2011; Welicky et al. 2017).

In South Africa, to date, a single study was performed to analyse the trophic relationship between an ectoparasite and its host fish inhabiting the Vaal Dam (Sures et al. 2019). Sures et al. (2019) demonstrated that the ectoparasitic monogenean, *Paradiplozoon ichthyoxanthon*, was enriched in ^15^N compared to two closely related fish species; Largemouth yellowfish (*Labeobarbus kimberleyensis*) and Smallmouth yellowfish (*Labeobarbus aeneus*). In addition to infection by *P. ichthyoxanthon*, Largemouth yellowfish is also host to the cestode *Schyzocotyle acheilognathi* (Bertasso and Avenant-Oldewage 2005; Retief et al. 2007). The aims of this study were therefore to compare stable isotope signatures of carbon and nitrogen within the *L. kimberleyensis*–*S. acheilognathi* system from the Vaal Dam in an attempt to better understand the trophic interactions between host and cestode. In addition, as the Largemouth yellowfish is also host to the monogenean, *P. ichthyoxanthon* comparisons between the stable isotope signatures detailed for *L. kimberleyensis*–*P. ichthyoxanthon* in Sures et al. (2019) and the host–cestode model in the present study were done to better understand the interactions of parasites which occupy different microhabitats within the same host organism and have different feeding strategies.

## Materials and methods

### Host and parasite collection

Collections of *Labeobarbus kimberleyensis* (*n* = 10) were conducted in the Vaal Dam, South Africa, around the University of Johannesburg (UJ) Island (26° 52′ 33.62″ S; 28° 10′ 25.76″ E) using gill nets (mesh size 45–170 mm) in March 2017. Fish were removed from the nets and immediately placed into 160-L plastic containers filled with aerated water from the dam. The fish were transported back to a field laboratory on UJ Island where weights and morphometric parameters (standard, fork and total length) were determined before dissection, for calculation of Fulton’s condition factor (*K*; Eq. 1) according to Heincke (1908).

1 $$ K=100\times \frac{SW}{TL^3} $$

with *SW:* as weight of the fish; *TL:* as total length.

The fish were euthanised by severing the vertebrae of the spinal cord, posterior to the head. Largemouth yellowfish were then dissected to expose the visceral organs; the intestines were removed and placed into Petri dishes. The intestines were opened using Dumont forceps to expose and collect *S. acheilognathi*. All cestodes were removed, counted and placed into 2-mL microcentrifuge tubes. Following removal of the intestines, muscle and liver tissue of the hosts were collected using plastic forceps and a ceramic knife and placed into 15-mL centrifuge tubes. All host and parasite samples were frozen at − 20 °C until stable isotope analysis. All procedures for the collection of fish and parasites from the Vaal Dam followed approval from the Faculty of Science Ethics Committee of the University of Johannesburg (Ethics reference number, 2016-5-03) and application of appropriate permits for the collection of sensitive species from the Gauteng Department of Agriculture and Rural Development (permit number, 009658).

Frozen host and parasite samples were freeze-dried using an Alpha 1–2 LDplus freeze drier (Martin-Christ Gefriertrocknungsanlagen, GmbH; Germany) for 48 h and then homogenised directly in the 2-mL microcentrifuge tubes using a stainless steel pestle. Analysis of stable isotopes in muscle and liver of the host was performed as muscle tissue has been mostly used for trophic position comparisons between organisms. The inclusion of liver tissue was due to the fact that cestodes rely on bile excreted from the liver for nourishment.

### Stable isotope analysis

For stable isotope ratio analysis of carbon (^13^C/^12^C) and nitrogen (^15^N/^14^N), triplicate samples of homogenised host tissues and parasites were weighed (0.2–0.7 mg) into tin capsules (4 × 6 mm) and folded. Folded capsules were placed into a PYRO Cube elemental analyser (EA; Elementar Analysensysteme, Langenselbold, Germany) coupled with an IsoPrime 100 isotope ratio mass spectrometer (IRMS; Elementar Analysensysteme) for the quantification of the isotope ratios of carbon and nitrogen in host and parasite tissues. Stable isotope ratios in tissue samples and internal standards were reported as *δ*-notation by Eq. (2) following Werner and Brand (2001) and Nachev et al. (2017).

2 $$ {\delta}^h{E}_{s, ref}=\frac{R{\left(\raisebox{1ex}{${h}_E$}\!\left/ \!\raisebox{-1ex}{${l}_E$}\right.\right)}_s}{R{\left(\raisebox{1ex}{${h}_E$}\!\left/ \!\raisebox{-1ex}{${l}_E$}\right.\right)}_{ref}}-1 $$

where $$ R{\left(\raisebox{1ex}{${h}_E$}\!\left/ \!\raisebox{-1ex}{${l}_E$}\right.\right)}_s $$: ratio of heavy and light isotopes for carbon and nitrogen in host and cestode tissues; $$ R{\left(\raisebox{1ex}{${h}_E$}\!\left/ \!\raisebox{-1ex}{${l}_E$}\right.\right)}_{ref} $$: ratio in reference materials.

Normalisation of the internal working standard acetanilide (AcAn) was done by two point calibration against the international standards, USGS40 and USGS 41 (International Atomic Energy Agency). The international standards were used as reference materials for the normalisation of isotope ratios determined in AcAn and host and cestode tissue samples.

### Statistical analyses

Normality of the data was tested using the Shapiro–Wilk test and as data was not normally distributed, non-parametric tests were used to compare differences in isotope signatures. Differences in isotope signatures between host and parasite were compared using the Kruskal–Wallis test. Post hoc comparison was performed using a pairwise comparison with Bonferroni correction.

Host–parasite discrimination factors (Δ^h^E) were estimated by subtracting isotope signatures in parasites from the signatures in host muscle and liver tissue (Eq. 3). The same equation was applied to assess isotope discrimination between the monogenean, *P. ichthyoxanthon* and *S. acheilognathi* (Δ^P^E (P.i-S.a)).

3 $$ {\Delta }^hE={\delta}^h{E}_{\mathrm{parasite}}-{\delta}^h{E}_{\mathrm{host}} $$

where *δ*^*h*^*E* are levels of ^13^C and ^15^N in host muscle and liver tissue and in cestodes. For comparison between parasites, the isotope signatures in the monogenean were subtracted from the isotope signatures in the cestodes.

Differences in trophic position (ΔTP) between host and cestode were calculated according to the following Eq. (4):

4 $$ \Delta  \mathrm{TP}=\frac{\left({\delta}^{15}{N}_{\mathrm{parasite}}-{\delta}^{15}{N}_{\mathrm{host}}\right)}{\mathrm{TEF}} $$

For estimation of the trophic position of cestodes relative to the fish host, the average trophic enrichment factor (TEF = 3.4‰) was used (Minagawa and Wada 1984). This was applied for isotope signatures between the parasite and muscle tissue, and not for the liver tissue of the host.

## Results and discussion

### Stable isotopes in host and parasite

**Fig. 1:**
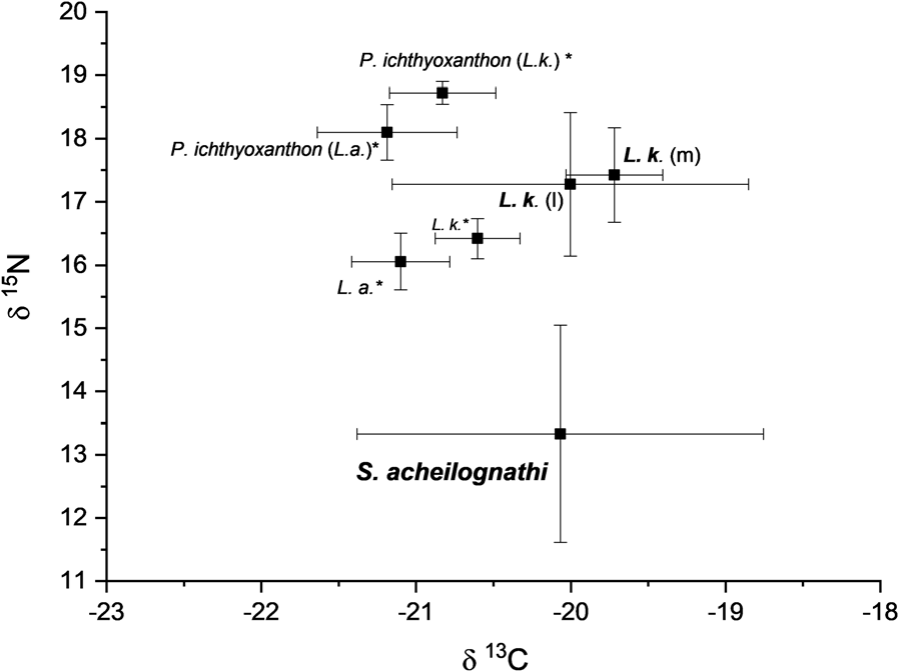
Mean isotope ratios and standard deviations for δ^15^N and δ^13^C in *Labeobarbus kimberleyensis* (*L.k*)*–Schyzocotyle acheilognathi* compared with data from Sures et al. (2019) (*) for *L. kimberleyensis–Paradiplozoon ichthyoxanthon* and *L. aeneus* (*L.a*)–*P. ichthyoxanthon* collected from the Vaal Dam. Isotope ratios for the muscle (m) and liver (l) from *L. kimberleyensis* are compared for the present study. Data from the present study are indicated as bold font

The cestode, *S. acheilognathi*, was found to be depleted in both ^15^N and ^13^C isotopes relative to the liver and muscle tissue of the host fish, *L. kimberleyensis* (Table 1 and Fig. 1). Differences in stable isotope composition between *S. acheilognathi* and the muscle and liver of *L. kimberleyensis* were significant for δ^15^N (Kruskal–Wallis test; *H* = 14.24; df = 2; *P* = 0.001) but not for δ^13^C (Kruskal–Wallis test; *H* = 2.08; df = 2; *P* = 0.354). This pattern is in line with other studies (Table 2) on adult and larval cestodes (Boag et al. 1998; Iken et al. 2001; Pinnegar et al. 2001; Deudero et al. 2002; Power and Klein 2004; Persson et al. 2007; Navarro et al. 2014; Behrmann-Godel and Yohannes 2015; McGrew et al. 2015) and acanthocephalans (Nachev et al. 2017) which have similarly been found to be depleted in ^15^N isotope compared to their hosts. Differences in ^13^C isotopes between host muscle tissue and cestode in the present study accounted for a difference of 1.2 ± 2.1‰ and therefore this difference is within the 1–2‰ enrichment factor reported in literature (DeNiro and Epstein 1981; Fry and Sherr 1984; Power and Klein 2004). However, this difference was not significant and is consistent with other studies where host and cestode parasites are relatively similar in terms of ^13^C enrichment (Power and Klein 2004), indicating that both host and parasite assimilate nutrients from similar sources (Power and Klein 2004).

**Table 1:** Fish morphometry and signatures of stable isotopes of carbon and nitrogen in the muscle and liver tissue of *Labeobarbus kimberleyensis* and cestode, *Schyzocotyle acheilognathi* compared with data presented by Sures et al. (2019) for isotope signatures in *L. kimberleyensis–Paradiplozoon ichthyoxanthon* from the Vaal Dam

	TL (cm)	W (kg)	k		δ^13^C	δ^15^N
*L. kimberleyensis*	42.4 (± 3.1)	0.7 (± 0.2)	0.9 (± 0.1)	Muscle	− 19.8 (± 0.4)	17.5 (± 0.8)
				Liver	− 20.9 (± 2.7)	15.8 (± 5.2)
*S. acheilognathi*	–	–	–		− 20.9 (± 2.2)	13.4 (± 1.8)
Δ^h^E (parasite-host)	–	–	–	Muscle	− 1.17 (± 2.14)	− 4.10 (± 1.11)
				Liver	− 0.04 (± 2.04)	− 2.47 (± 5.92)
L. kimberleyensis*	35.8 (± 6.7)	0.43 (± 0.2)	1.0 (± 0.2)	Muscle	− 20.61 (± 0.28)	16.42 (± 0.32)
P. ichthyoxanthon*	–	–	–		− 20.83 (± 0.35)	18.73 (± 0.18)
Δ^h^E (parasite-host)*	–	–	–		− 0.22	− 2.31
Δ^P^E (P.i-S.a)	–	–	–		0.07	5.33

*Data from Sures et al. 2019

**Table 2:** Summary of the isotopic differences (Δ^h^E (parasite-host)) for stable isotopes of nitrogen (Δ^15^N) and carbon (Δ^13^C) between hosts and cestodes of varying ontogenetic development from different environments

Author	Host	Macroenvironment	Parasite	Developmental stage	Microhabitat	Δ^h^E (parasite-host)	
						Δ^15^N	Δ^13^C
Boag et al. 1998	*Oryctolagus cuniculus*	Terrestrial	*Cittotaenia denticulata*	Adult	Intestine	− 2.10	− 1.32
			*Mosgovoyia pectinata*	Adult	Intestine	− 3.10	− 3.66
Pinnegar et al. 2001	*Gasterosteus aculeatus*	Limnetic	*Schistocephalus solidus*	Plerocercoid	Peritoneal cavity	− 2.41	− 0.14
Deudero et al. 2002	*Rutilus rutilus*	Limnetic	*Ligula intestinalis*	Plerocercoid	Peritoneal cavity	− 1.42	− 0.43
Power and Klein 2004	*Gasterosteus aculeatus*	Limnetic	*Schistocephalus solidus*	Plerocercoid	Peritoneal cavity	− 1.35	0.13
	*Salvelinus fontinalis*	Estuarine	*Eubothrium salvelini*	Adult	Intestine	− 2.08	0.01
	*Gadus ogae*	Marine	*Pyramicocephalus phocarum*	Plerocercoid	Muscle*	− 4.43	0.52
Neilson et al. 2005	*Oryctolagus cuniculus*	Terrestrial	*Mosgovoyia pectinata*	Adult	Intestine	− 1.50	− 5.50
			*Cittotaenia denticulata*	Adult	Intestine	− 2.70	− 1.90
Persson et al. 2007	*Salmo salar*	Marine	*Eubothrium crassum*	Adult	Intestine	− 2.45	1.61
Sánchez et al. 2013	*Artemia parthenogenetica*	Brackish	*Flamingolepis liguloides*	Cysticercoid	Haemocoel	0.24	2.82
Navarro et al. 2014	*Chimaera monstrosa*	Marine	*Gyrocotyle urna*	Adult	Gastrointestinal tract (stomach, spiral valve)	− 3.33	− 1.32
Behrmann-Godel and Yohannes 2015	*Perca fluviatilis*	Limnetic	*Triaenophorus nadulosus*	Plerocercoid	Liver	− 0.79	− 2.17
Eloranta et al. 2015	*Gasterosteus aculeatus*	Limnetic	*Schistocephalus solidus*	Plerocercoid	Peritoneal cavity	− 2.20	− 0.40
	*Pungitius pungitius*		*Schistocephalus pungitii*	Plerocercoid	Peritoneal cavity	− 2.00	0.30
McGrew et al. 2015	*Canis lupus*	Terrestrial	*Taenia krabbei*	Adult	Intestine	− 2.20	− 2.00
			*Taenia hydatigena*	Adult	Intestine		

The host fish muscle tissue was enriched by 4.1 ± 1.1‰ in the heavier nitrogen isotope with respect to the parasite, indicating that the host occupies a higher trophic position than the parasite (based on equation for ΔTP (4)). Liver tissue was similarly enriched in the heavier nitrogen isotope by 2.5 ± 5.9‰. For carbon isotopes, comparison with liver tissue of Largemouth yellowfish hosts showed cestodes were depleted in ^13^C by 0.04 ± 2.04‰ relative to the host and therefore, host liver and *S. acheilognathi* shared similar δ^13^C signatures. These results are in line with the mode of nutrient acquisition observed in other endohelminths such as cestodes and acanthocephalans (Nachev et al. 2017). Cestodes lack a digestive system and are unable to metabolise complex molecules (Smyth and McManus 1989). As a result, these parasites acquire pre-digested nutrients from the hosts’ intestine but also reprocessed compounds derived from the hosts’ metabolites in bile salts. These compounds, which are assimilated across their highly specialised tegument, are depleted in heavier isotopes (Doucett et al. 1999; Nachev et al. 2017). This ultimately leads to endohelminths, such as cestodes, being depleted in heavier isotopes compared to their hosts (Nachev et al. 2017). Similarities in δ^13^C in host liver and cestodes confirm that *S. acheilognathi* derives nutrients from metabolites released from the liver of the host and not through direct feeding on host tissue. Thus, cestodes as well as acanthocephalans might be assumed to have a somewhat more commensalistic and scavenger-type feeding strategy as they do not compete directly with the host for the same food source and do not feed actively on host tissues.

Large standard deviations in stable isotope levels were observed in the liver tissue of the host and cestodes. This variability could be related to the metabolic activity of the host and parasite tissues as well as variability in the feeding biology of the host fish (Neilson et al. 2005; Behrmann-Godel and Yohannes 2015; Yohannes et al. 2017). Behrmann-Godel and Yohannes (2015) suggest that variability in isotope levels of intestinal helminths is likely the result of the nutrient component they are exposed to in the intestine. Thus the variability in stable isotopes observed for *S. acheilognathi* may relate to shifts in the host diet. Yohannes et al. (2017) further indicated that higher variability in stable isotope levels of different organs of the perch (*Perca fluviatilis*) was related to the metabolic activity of the different organs. In their study, pike liver had higher isotope turnover rates than blood and muscle tissue. Comparison of host fish muscle and liver showed higher standard deviations in liver tissue than in muscle. Yohannes et al. (2017) related this to the higher metabolic activity of the liver compared with the other tissues analysed in their study and thus the same may apply in the present study.

As *L. kimberleyensis* was also often parasitised by the monogenean parasite, *P. ichthyoxanthon*, the results from the present analysis were compared to those performed by Sures et al. (2019). *Labeobarbus kimberleyensis* collected in the current study were of a larger size and more enriched in ^15^N than specimens analysed by Sures et al. (2019), but the opposite was observed for ^13^C signatures which were higher in smaller hosts. This finding can be related to the larger size of the yellowfish collected in the present study compared to those collected by Sures et al. (2019). As *L. kimberleyensis* grow, they undergo a dietary shift from a predominantly omnivorous diet to a predatory one where they feed on smaller fish specimens, even including juvenile yellowfish (Skelton 2001; Sures et al. 2019). This change in food source generally occurs when *L. kimberleyensis* attain a fork length of approximately 300 mm (Skelton 2001). Fish hosts collected in the present study were larger than 300 mm and therefore had likely undergone a dietary shift which had led to the observed shift in the ^15^N signatures. Comparison between hosts from each study showed that the larger *L. kimberleyensis* in the present study (present study, 700 g; Sures et al. 2019, 430 g) were more enriched in δ^15^N (present study, 17.5‰; Sures et al. 2019, 16.42‰) than Largemouth yellowfish analysed by Sures et al. (2019), but δ^13^C signatures did not differ within the sample from each study.

Comparison of stable isotope signatures between *P. ichthyoxanthon* and *S. acheilognathi* (Table 1; Δ^P^E (P.i-S.a)) showed that ^15^N enrichment in *P. ichthyoxanthon* was 5.3‰ greater than in *S. acheilognathi* which accounts for a difference equal to almost two trophic positions. Differences in δ^13^C between the parasites accounted for 0.07‰, indicating that both parasites infecting *L. kimberleyensis* derive nourishment from the host and thus share a common food source (Table 1). Differences in stable isotope enrichment in *P. ichthyoxanthon* and *S. acheilognathi* can be related to the method of acquiring nutrients by each parasite. *Paradiplozoon ichthyoxanthon* behaves similarly to a predator and derives nutrients by feeding directly on host blood from the gills. *Schyzocotyle acheilognathi* on the other hand does not feed on host intestinal epithelium but rather by assimilation of metabolites released as components of bile by the host, as mentioned above. Nachev et al. (2017) observed a similar difference in isotope signatures between the nematode, *Eustrongylides* sp., and the acanthocephalan *Pomphorhynchus laevis*, which both infect *Barbus barbus*. The nematode was more enriched in ^15^N relative to the acanthocephalan, and similarly to *P. ichthyoxanthon*, feeds on host tissue. In contrast, *P. laevis* was depleted in ^15^N, and similarly to *S. acheilognathi*, assimilates by-products from the host across the tegument.

Overall, only a handful of studies have analysed differences in stable isotopes between hosts and cestodes (Table 2). From these studies, it appears that adult and larval stages of cestodes are variably depleted in nitrogen and carbon isotopes relative to their hosts. This variability in the enrichment of cestodes with either isotope may be related to the microhabitat of the parasite and the feeding strategy adopted by this group of parasites. From the enrichment factors of stable carbon and nitrogen isotopes, it is clear that both adult and larval cestodes absorb metabolites from the host and not through active feeding on the host. Most developmental stages of cestodes are depleted in ^15^N relative to the host organism indicating that regardless of the developmental stage, cestodes feed through absorptive routes. Adult stages were isotopically depleted relative to the hosts and, similarly, in the case of *S. acheilognathi* from the present study, this can be related to the food source utilised by this developmental stage as being isotopically depleted. Plerocercoid larvae infecting the peritoneal cavity were mostly depleted in ^15^N compared to their hosts but fractionation patterns of δ^13^C are mixed compared to their hosts (Pinnegar et al. 2001; Deudero et al. 2002; Power and Klein 2004; Eloranta et al. 2015). However, *Flamingolepis liguloides* cysticercoid larvae infecting the haemocoel of the host, *Artemia parthenogenetica*, were isotopically enriched in both ^15^N and ^13^C relative to their hosts (Sánchez et al. 2013). The enrichment of larval stages with particular isotopes may point to differences in transmission pathways between systematically different groups of cestodes such as cyclophyllids and bothriocephalids, which have different larval stages, i.e. cysticercoides and procercoids and plerocercoids, respectively.

## Conclusion

Results of the present study indicate that the cestode, *S. acheilognathi*, was isotopically depleted compared to the host fish *L. kimberleyensis*. This pattern corroborated previous studies on cestodes and can be linked to the source of nutrition being depleted in the heavier isotopes. This results in cestodes occupying at least 0.1–1.3 trophic positions below host. However, this varies between adult and larval stages. Additionally, this indicates that unlike other intestinal parasites such as adult nematodes, cestodes have adopted a somewhat commensalistic and scavenger-type feeding strategy and therefore do not compete directly with the host for nutrients. Stable isotope analysis not only serves as a useful tool for delineating the relationship between different life stages of parasites and hosts in the food web but may also be implemented as a means of tracing transmission pathways of larval parasites between intermediate and definitive hosts. To attain this, additional and intensive comparisons between the nutritional sources of hosts and parasites are required in future studies.
